# Cross-Cultural Medicine in the Middle East at the Start of the 21st Century: Where East and West Meet

**DOI:** 10.1100/tsw.2006.346

**Published:** 2006-07-06

**Authors:** Jacob Urkin, Mohammed Morad, Joav Merrick, Yaakov Henkin

**Affiliations:** ^1^ Clalit Health Services, Beer-Sheva, Israel; ^2^ Pediatric Primary Care Unit, Ben Gurion University of the Negev, Beer-Sheva, Israel; ^3^ Faculty of Health Sciences, Ben Gurion University of the Negev, Beer-Sheva, Israel; ^4^ Division of Pediatrics, Soroka University Medical Center, Israel; ^5^ National Institute of Child Health and Human Development, Faculty of Health Sciences, Ben Gurion University of the Negev, Beer-Sheva, Israel; ^6^ Center for Multidisciplinary Research in Aging, Faculty of Health Sciences, Ben Gurion University of the Negev, Beer-Sheva, Israel; ^7^ Office of the Medical Director, Division for Mental Retardation, Ministry of Social Affairs, Jerusalem, Israel; ^8^ Department of Cardiology, Soroka University Medical Center, Israel; ^9^ Medical School for International Health in collaboration with Columbia University Medical Center, Beer-Sheva, Israel

**Keywords:** medicine, culture, international medicine, education, Bedouin, Israel

## Abstract

The “global village” has resulted in the need to tackle cross-cultural issues in the medical school curriculum. The southern region of Israel (the Negev) provides a unique opportunity to study the interaction between medicine and culture. The Negev population is a multicultural society, with Bedouin Arabs comprising almost a fifth of its population. This imposes tremendous challenges to the medical establishment in the region and serves as a “cross-cultural laboratory” for educating medical students in global health issues. Both the traditional Israeli medical school track, as well as the newly established Medical School for International Medicine, incorporate studies of cross-cultural issues in various forms and to different degrees. Studies suggest that the exposure of students to international medical experiences increases their cross-cultural sensitivity and knowledge. We feel that in a region characterized by such ethnic diversity, all medical schools should adopt cross-cultural studies as an integral part of their curriculum.

## INTRODUCTION

Fast global transportation, communication, and immigration have brought many physicians to face cross-cultural and global health problems they have not been trained to address. Social, economic, political, and technological changes facilitate the spread of infectious diseases between communities. Immigration, armed conflict, draught, and tourism are examples of the forces that lead to the movement of large numbers of people from one place to another, where provision of medical services becomes a cross-cultural mission.

Cross-cultural challenges are also encountered within societies with different cultural backgrounds. Import of new technologies and ideas through television and the Internet can challenge a traditional society and impose modifications of medical services. This also happens when physicians educated in “western medicine” encounter patients who believe in traditional or alternative medicine[1].

When a large proportion of a population endures radical changes in values and standards of living over a short period of time, new subcultures evolve. Part of the change is reflected in the attitude of its members towards medicine. This process occurs throughout the world, facilitated by mass media and the spread of information technology, such as television and electronic media. It is not a surprise anymore to observe satellite dishes in remote villages. However, the changes in values and attitudes of people are much slower than the change in lifestyle and standard of living. As a result, medical education requires continuous adaptation to enable graduates to act as competent health providers to patients of increased cultural diversity[2,3].

## SOUTHERN ISRAEL AS AN EXAMPLE OF CULTURAL DIVERSITY

The southern region of Israel (the Negev) provides a unique opportunity to study the interaction between medicine and culture. Geographically, it consists of over 13,000 km$^2$ of land in the form of an inverted triangle framed by the borders of Jordan and Egypt, with its southernmost tip at Eilat.

The region, formerly a desert, was an important source of grain for the Roman Empire, but after the collapse of the Roman Empire, only Bedouin tribes tended their sheep and goats in the region. At the end of the 19th century, the Turkish Empire constructed the town of Beer-Sheva (currently the capital of the Negev) in its present location. In 1917, the British Army conquered the city and in 1948, the region became part of the state of Israel[4]. In addition to Beer-Sheva, the region currently comprises several towns, villages, and other agricultural settlements.

For centuries, the area has been home to the Bedouins, who have increasingly given up their nomadic lifestyle and settled in permanent homes. Since the establishment of the state of Israel, Jewish immigrants from all around the world, particularly North Africa, Eastern Europe, and America, have also come to live in the Negev. Additional important immigration groups have come from India and Ethiopia. A small group of “Black Hebrews” from Chicago began to arrive in Israel in the late 1960s and settled in the Negev town of Dimona, living under unique rules of conduct and cultural activities. During the 1990s, a large immigration from Eastern Europe also increased the Negev population. Thus, the Negev region embraces a multicultural society, which requires tremendous challenges to the medical establishment in the region and can serve as a “cross-cultural laboratory” for the education of medical students in global health issues.

### The Bedouin Populations in Southern Israel as a Unique Model for Teaching Cross-Cultural Medicine

A large Bedouin population resides in the Negev region and comprises approximately 18% of the population in the region. In regard to health, the Bedouin population can be characterized by many cross-cultural components. More than half of this population has changed its nomadic lifestyle as shepherds to residents in permanent settlements, though approximately 40% still live in settlements that are not formally recognized by the Israeli government. A dramatic drop in the infant mortality rate in the past 2 decades has resulted in larger families and a rapid expansion of the size of the Bedouin population[5,6]. Since this was not accompanied by an increase in appropriate occupation opportunities, the rate of unemployment is high and many of the Bedouin families are poor. Although traditional medicine still exists, its popularity is declining as western medicine becomes more available and accessible, especially within the recognized settlements.

Exposure to the western lifestyle is noticeable, especially among the younger generations. More Bedouin youngsters seek university education and are thus exposed to non-Bedouin culture. This is reflected by changes in lifestyle and food consumption, accompanied by a rise in western ailments, such as obesity, diabetes, and cardiovascular disease[7]. Although some still choose to retain the more orthodox Arab lifestyle, many parents and children are drifting apart, as the traditional values of the Bedouin family become harder to maintain.

Epidemiological studies suggest higher rates of certain acute and chronic diseases in the Bedouin population compared to Jews[8,9,10]. Certain features of the Bedouin tradition have special implications to their health. Consanguineous marriage is very common and is one of the major reasons for the high rate of genetic disorders, as well as infant mortality and morbidity in this population[11]. Although all citizens are covered by a national health insurance with free medical services, socioeconomic and cultural factors influence the use of these services[12,13]. Some excess of disease in Bedouins is attributed to a knowledge gap in regard to health maintenance and prevention, some to the low standards of living conditions, and some to the traditional acceptance of disease as a faith that is not influenced by one’s behavior[12]. High rates of cigarette smoking, obesity, and diabetes in adults[14], and iron deficiency and excess of household injuries in children[15,16], are examples of preventable medical problems that are on the increase in the Bedouin population in southern Israel. Intervention programs that were sensitive to the special needs of the Bedouin population were designed by Bedouin health educators, but achieved only modest implementation as emotional and traditional factors did not succumb easily to the western kind of logic[17].

The background of the medical personnel who work with the Bedouin community is diverse. It includes Arab and Jewish health workers who were educated in Israel, as well as in Eastern and Western Europe. For the non-Arab health workers, barriers exist in the form of language in addition to the unfamiliarity with local tribal tradition of their patients. In addition, many Bedouin patients still seek medical advice from traditional healers or second opinions from Palestinian physicians who usually are not able to communicate with the Israeli health providers.

## MEDICAL EDUCATION IN SOUTHERN ISRAEL

The first medical school at Ben Gurion University (BGU) of the Negev was established in 1974 in an attempt to integrate medical care with medical education, provide better community orientation of graduates, and improve the health care availability in the region[2,3,18]. The program is designed as a 6-year curriculum followed by a rotational internship. From its inception, the program emphasized early clinical studies[19]. The first teaching module starts in the first year of medical school, where communication with patients is taught and practiced. In addition to the skills of medical interviewing, the students receive the basic knowledge and attitude regarding cross-cultural issues. Respecting values that are different from one’s background and recognizing nonverbal messages are examples of the issues that are discussed. Interaction with the Bedouin culture continues during the clinical years, as a large proportion of the patients at Soroka University Medical Center (the major teaching center of the faculty) are Bedouins, both in inpatient as well as outpatient facilities. This interaction is especially prominent in the delivery rooms and the pediatric wards, where approximately 50% of newborns and hospitalized children are Bedouin.

In 1998, a new medical school was established at BGU in collaboration with Colombia University in New York. This Medical School for International Health (MSIH) is specifically dedicated to the training of physicians in aspects of international health and medicine (IHM). The goal of the new 4-year MD program, taught in English and designated for non-Israeli citizens, is to graduate physicians with special skills in IHM and the ability to prevent, diagnose, and treat illness in cultures other than their own. The curriculum emphasizes the export of modern western medicine to developing countries[20,21], as well as what western medical students can learn from working and living in traditional cultures[22]. It is unique in its focus on cross-cultural aspects of medicine and goes beyond the individual orientation of traditional medical training to focus on the health needs of populations. In addition to the standard education for the MD degree, graduates acquire knowledge about the impact of economic, sociopolitical, cultural, environmental, and policy factors on the health of individuals and populations. An introductory course in IHM is given in the first year of medical school and special seminars in IHM are given throughout the 4 years[23,24]. Early clinical exposure, adopted from the medical school program for Israelis, includes ample interactions with Bedouin, Ethiopian, and other ethnic cultures in the community as well as institutional settings. In the third year, there is a student workshop in cross-cultural communication that uses small group teaching and specially trained standardized patients[25]. In the fourth year, students participate in a 2-month clinical clerkship in IHM[23]. While most of these clerkships are held in various third-world countries, some students choose to remain in Israel and participate in a specifically designed rotation in Arabic and Ethiopian communities. In addition to their formal studies, many of the students participate in voluntary activities such as teaching English to Bedouin children and working in foreign-workers' health clinics.

## BEDOUINS IN MEDICAL PROFESSIONS

School attendance is mandatory and free of charge for all Israeli children, and virtually all Bedouin children attend school, but the expenditure of books, school uniforms, etc. are an extra economic burden and the drop-out rate in this population is high beyond the eighth grade, especially for girls. The number of high school graduates who are eligible for university education is low in comparison to the Jewish population and to other Arab and non-Arab minorities. This situation results in a negligible number of Bedouins admitted to Israeli medical schools and forces others to study medicine in East Europe, where the admission criteria are less competitive. There are currently about 80 Bedouin physicians in the Negev region, but less than 10% graduated from Israeli universities.

Bedouin women who wish to acquire education in the medical professions have to overcome even greater barriers. Traditional Bedouin culture does not allow women to "mingle" with strangers without a chaperon. Going to the university involves the omission of this tradition and, in many cases, involves a change in the dress code and social relationships. Bedouin female physicians are scarce. Paramedical professions, such as nursing, are also of special challenge to Bedouin youth. Nursing has low prestige in the Bedouin community and it takes time to change that attitude. Those who graduate from nursing school usually return to their communities and integrate in the ambulatory health service. Their help in teaching medical students and promoting communication between the population and the non-Arab speaking staff in the clinics is invaluable.

Working in the medical profession often involves exposure to privacy issues of patients and to the privacy of their body. Cultural barriers regarding these issues often create obstacles for Bedouin medical professionals, including students. Some Bedouin patients are reluctant to be examined by a Bedouin professional, especially if the latter comes from a "rival tribe". Some will not disclose important information, as this may result in consequences on the basis of "honor of the family". Sensitivity to these issues is discussed with the students of the medical schools and methods to overcome them are explored.

## IS THERE EVIDENCE THAT CROSS-CULTURAL EDUCATION HAS AN IMPACT ON STUDENT CULTURAL SENSITIVITY?

Integrating cross-cultural aspects of medicine into the already overloaded curriculum of medical education requires a heavy investment by both faculty and students. Only a handful of studies have attempted to perform a systematic evaluation of the benefit of such endeavors[23,24,26]. A clinical elective in a developing country preceded by a preparatory course in IHM increased students' cultural sensitivity, enhanced their communication and clinical skills, and resulted in a strong preference to work with underserved populations and engage in community service activities after graduation[26,27]. A

survey of student attitude and knowledge of students in the MSIH at BGU showed that the students were able to increase their knowledge of the subject significantly and retain their positive attitudes toward IHM during the course of medical school[23,24]. These students had more positive attitudes towards IHM than students on the Israeli medical track as well as other U.S. medical schools.

## CONCLUSIONS

Cross-cultural medicine is of growing importance and should be taught in all medical schools around the world[28]. An increasing number of medical schools have become aware of this potential and have included elements of IHM and cross-cultural issues in their medical curriculum, often in the form of international elective experiences[28,29,30]. Cross-cultural sensitivity is particularly important in regions of the world, such as Israel, where a strong diversity of the population exists. The Bedouin population in southern Israel exemplifies the complexity of the issue and serves the medical school as a model for educating its students in such issues. We believe that medical schools should incorporate certain elements of the MSIH program, such as IHM modules and the cross-cultural workshop. Better acquaintance with minority cultures has the potential to improve medical care and resolve tensions that naturally exist in such diverse populations.

## BIOSKETCHES

**Jacob Urkin MD, MPH**, is a lecturer in pediatrics and director of the Primary Care Unit, Division of Community Health and director of the Maternal and Child program of the MPH program at the Faculty of Health Sciences, Ben Gurion University of the Negev, Beer-Sheva, Israel. Major academic interests are primary care, community pediatrics, well baby care services, school health, and medical education. E-mail: jacobur@clalit.org.il

**Mohammed Morad, MD**, is specialist in family medicine, lecturer in family medicine affiliated with the Department of Family Medicine, Division of Community Health, National Institute of Child Health and Human Development and Center for Multidisciplinary Research in Aging, Faculty of Health Sciences, Ben Gurion University of the Negev and the medical director of a large area clinic in the city of Beer-Sheva operated by the Clalit Health Services. Dr. Morad's publications include those on Bedouin health, health aspects, spiritual health, and aging in persons with intellectual disability, and he is a presenter on topics such as health policy and services for the disadvantaged at national and international conferences. E-mail: morad@bgu.ac.il

**Joav Merrick, MD, MMedSci, DMSc,** is professor of child health and human development affiliated with the Center for Multidisciplinary Research in Aging, Zusman Child Development Center, Division of Pediatrics and Community Health at the Ben Gurion University, Beer-Sheva, Israel; the medical director of the Division for Mental Retardation, Ministry of Social Affairs, Jerusalem; and the founder and director of the National Institute of Child Health and Human Development. Dr. Merrick has numerous publications in the field of child health and human development, rehabilitation, intellectual disability, disability, health, welfare, abuse, advocacy, quality of life, and prevention and received the Peter Sabroe Child Award for outstanding work on behalf of Danish Children in 1985 and the International LEGO-Prize ("The Children's Nobel Prize") for an extraordinary contribution towards improvement in child welfare and well being in 1987. E-mail: jmerrick@internet-zahav.net. Website: www.nichd-israel.com

**Yaakov Henkin, MD,** is a cardiologist at the Soroka University Medical Center; senior lecturer at the Faculty of Health Sciences, Beer-Sheva; and associate director for Academic Affairs, Medical School for International Health at Ben Gurion University in collaboration with Columbia University Medical Center, New York. Research interests and publication topics include preventive cardiology, lipoproteins, coronary risk factors, and nutrition. E-mail: yaakovh@bgu.ac.il. Website: http://fohs.bgu.ac.il/people/PDetails.asp?StaffID=11563
